# The Wsc1p Cell Wall Signaling Protein Controls Biofilm (Mat) Formation Independently of Flo11p in *Saccharomyces cerevisiae*

**DOI:** 10.1534/g3.113.006361

**Published:** 2013-12-06

**Authors:** Neha Sarode, Sarah E. Davis, Robert N. Tams, Todd B. Reynolds

**Affiliations:** Department of Microbiology, University of Tennessee, Knoxville, Tennessee 37996

**Keywords:** Flo11, biofilm, yeast, Wsc1p, Skn7

## Abstract

*Saccharomyces cerevisiae* strains of the ∑1278b background generate biofilms, referred to as mats, on low-density agar (0.3%) plates made with rich media (YPD). Mat formation involves adhesion of yeast cells to the surface of the agar substrate and each other as the biofilm matures, resulting in elaborate water channels that create filigreed patterns of cells. The cell wall adhesion protein Flo11p is required for mat formation; however, genetic data indicate that other unknown effectors are also required. For example, mutations in vacuolar protein sorting genes that affect the multivesicular body pathway, such as *vps27∆*, cause mat formation defects independently of Flo11p, presumably by affecting an unidentified signaling pathway. A cell wall signaling protein, Wsc1p, found at the plasma membrane is affected for localization and function by *vps27∆*. We found that a *wsc1∆* mutation disrupted mat formation in a Flo11p-independent manner. Wsc1p appears to impact mat formation through the Rom2p-Rho1p signaling module, by which Wsc1p also regulates the cell wall. The Bck1p, Mkk1/Mkk2, Mpk1p MAP kinase signaling cascade is known to regulate the cell wall downstream of Wsc1p-Rom2p-Rho1p but, surprisingly, these kinases do not affect mat formation. In contrast, Wsc1p may impact mat formation by affecting Skn7p instead. Skn7p can also receive signaling inputs from the Sln1p histidine kinase; however, mutational analysis of specific histidine kinase receiver residues in Skn7p indicate that Sln1p does not play an important role in mat formation, suggesting that Skn7p primarily acts downstream of Wsc1p to regulate mat formation.

Biofilms are the preferred modes of growth for many microorganisms in nature. A biofilm is a community of cells that aggregates and colonizes a foreign surface (Blankenship and Mitchell 2006). A major advantage of forming a biofilm is protection from the constant barrage of stresses that organisms are exposed to in the environment (Blankenship and Mitchell 2006). *Saccharomyces cerevisiae* is an attractive fungal model system to study genes important for biofilm formation because it is capable of forming an elaborate multicellular biofilm (hereafter referred to as a mat) on semi-solid agar (0.3%) and is easily manipulated genetically. The *S. cerevisiae* mat grows into a wheel-like structure that can be visually differentiated into a central wrinkled hub consisting of water channels, some of which resemble spokes of a wheel, all surrounded by a growing smooth rim (Reynolds and Fink 2001). These mats resemble biofilms that are called velum or flor that certain yeast strains form on the surfaces of sherry wines during fermentation (Fidalgo *et al.* 2006). It is possible that biofilm formation plays a role in the survival of yeasts during natural fermentation in rotting fruit, and a better understanding of the pathways that control yeast form biofilms may be useful for developing biofilm-based ethanol fermentation for bioethanol production.

In addition, *S. cerevisiae* is a useful model for understanding fungal pathogens like *Candida albicans* (Berman and Sudbery 2002). Biofilms by pathogenic fungi like *C. albicans* are a serious problem in clinical settings, where they colonize implanted medical devices and establish infection in immunosuppressed patients (Pfaller and Diekema 2007; Arendrup 2010). Work on *S. cerevisiae* biofilms may help the advancement of studies of biofilm formation in *C. albicans*.

The ability to form biofilms in fungi is largely dependent on various GPI-anchored adhesion proteins of the flocculin (FLO) family that are localized to the cell wall. Flo11p is the only FLO protein expressed in mats formed by the *S. cerevisiae* strains derived from L6906 (∑1278b background) (Halme *et al.* 2004; Reynolds *et al.* 2008), and mat formation is dependent on Flo11p. However, there are pathways that regulate mat formation by controlling unidentified cell wall effectors that act parallel to Flo11p. For example, a number of vacuolar protein sorting (VPS) genes affect mat formation through pathways that directly affect Flo11p or act parallel to this pathway. The pathway that acts parallel to the Flo11p-dependent pathway is called the biofilm pathway, and it requires an intact and fully functional multivesicular body (MVB) pathway involving the endosome (Sarode *et al.* 2011). Based on our previous results, it was hypothesized that MVB pathway mutants affect mat formation by mislocalizing an important component of the biofilm pathway, leading to perturbation of the cell wall and ultimately to defects in mat formation.

There are a number of pathways that affect the cell wall. One that has components affected by the MVB pathway is the cell wall integrity pathway (CWI). The CWI pathway consists of several signaling modules that include a family of single transmembrane domain sensors (Wsc1p is the main sensor for the wall), a Rho-type GTPase and its guanylate exchange factor (GEF) (*i.e.*, Rho1p and Rom2p, respectively), a protein kinase C homolog (Pkc1p), and a MAP kinase (MAPK) cascade (Bck1p-Mkk1/Mkk2p-Mpk1p) (Gustin *et al.* 1998; Levin 2005) (Supporting Information, Figure S1).

Activation of the CWI pathway has pleiotropic effects on cell wall repair and biogenesis. The main functions of the CWI pathway are maintenance of the highly dynamic cell wall structure by sensing signals (*i.e.*, damage attributable to physical or environmental agents, pheromones, cues to divide, and others) and relaying them downstream, leading to activation of appropriate genes that encode proteins that remodel the cell wall. In addition to Pkc1p and the CWI–MAPK cascade, Rho1p also directly regulates the Fks1p β-1,3-glucan synthase and the Skn7p transcription factor, both of which affect the cell wall. Data are presented revealing that components of the CWI pathway, including the Wsc1p receptor but excluding the CWI–MAPK cascade, are required for mat formation and therefore may comprise a part of, or the entire, biofilm signaling pathway.

## Materials and Methods

### Strains, media, and growth conditions

All strains used in this study belong to the yeast strain background ∑1278 and were derived from the strain L6906 (Reynolds and Fink 2001; Reynolds, *et al.* 2008) (Table 1). The *wsc1Δ* and *skn7Δ* mutants were created by transforming in the KanMX4 disruption cassette amplified by PCR (Longtine *et al.* 1998) from the genomes of the *wsc1∆* and *skn7∆* mutants pulled out from the respective mutants in a whole-genome deletion collection created in the Σ1278 background by Ryan *et al.* (2012) in the laboratory of Charles Boone at the University of Toronto. The Wsc1-GFP strain was created as follows: GFP-HIS3MX6 cassette from pFA6a-GFP-HIS3MX6 (Longtine *et al.* 1998) was PCR-amplified and inserted by homologous recombination just 5′ to the stop codon of the *WSC1* gene on the chromosome to create the strain NY87. The WSC1-GFP cassette from NY87 was PCR-amplified from the genome and inserted into pRS306 (Sikorski and Hieter 1989) using primers NSO46 and NSO47 to incorporate flanking *Xba*I and *Hin*dIII sites. This plasmid was integrated into the NY68 *wsc1∆* mutant at the *wsc1∆* locus to create strain NY236. The plasmid was also used as a template for site-directed mutagenesis of the *WSC1* cytoplasmic tail DNA. These mutant versions of the plasmid were also integrated into the NY68 strain. Primers for PCR reactions are listed in Table 2. Transformations were performed by the standard lithium acetate transformation method (Styles 2002). All strains were maintained on standard yeast extract-peptone-dextrose (YPD) media (Styles 2002) or YPD plates containing 250 µg/ml G418 or on minimal media lacking histidine (Styles 2002). Strains grown on low-agar plates (YPD with 0.3% agar) (Reynolds and Fink 2001) were maintained for 5 days at 25° and were used for overlay adhesion assays and immunofluorescence staining.

**Table 1:** Strains used in this study

**Strain**	**Genotype**	**Reference or Source**
TRY181	*MAT***a** *ura3-52 his3*::*hisG FLO11*::HA^30,1015^	Sarode *et al.* (2011)
NY68	*MAT***a** *ura3-52 his3*::*hisG FLO11*::HA^30,1015^ *wsc1*::kanMX6	This study
NY78	*MAT***a** *ura3-52 his3*::*hisG FLO11*::HA^30,1015^ *skn7*::kanMX6	This study
NY270	*MAT***a** *ura3-52 his3*::*hisG FLO11*::HA^30,1015^ *rom2*::kanMX6	This study
NY87	*MAT***a** *ura3-52 his3*::*hisG FLO11*::HA^30,1015^ WSC1-GFP-HIS3MX6	This study
NY236	*MAT***a** *ura3-52 his3*::*hisG FLO11*::HA^30,1015^ *wsc1Δ*::WSC1-GFP-HIS3MX6	This study
NY245	*MAT***a** *ura3-52 his3*::*hisG FLO11*::HA^30,1015^ *wsc1Δ*::WSC1-Y303A-GFP-HIS3MX6	This study
NY249	*MAT***a** *ura3-52 his3*::*hisG FLO11*::HA^30,1015^ *wsc1Δ*::WSC1-L369A-V371A-GFP-HIS3MX6	This study
NY251	*MAT***a** *ura3-52 his3*::*hisG FLO11*::HA^30,1015^ *wsc1Δ*::WSC1-S19A-S20A-GFP-HIS3MX6	This study
NY254	*MAT***a** *ura3-52 his3*::*hisG FLO11*::HA^30,1015^ *wsc1Δ*::WSC1-Y303A-L369A-V371A-GFP-HIS3MX6	This study

**Table 2:** Primers used in this study

**Name**	**Purpose**	**Sequence**
TRO693	Disrupt *WSC1*	TTTTCGAAGCGAAAGCGAGA
TRO694	Disrupt *WSC1*	TTAATGTTCCTCGTTACTTCCAG
NSkn7F	Disrupt *SKN7*	CAAGATTGAAAGTGCTTCCAGG
NSkn7R	Disrupt *SKN7*	CGCATACTAAATTACTGTGTCTGT
TRO783	Insert GFP-HIS3MX6 from pFA6a-GFP-His3MX6	CAGGAGGGAAAAACAACGTTTTAACAGTGGTCAATCCAGACGAAGCTGAT
TRO784	Insert GFP-HIS3MX6 from pFA6a-GFP-His3MX6	AGACTTGCTTGGCAATAGTTTAAGAATATAATAATTTTTTTTGGGTTTCTTCA
TRO369	Reverse primer to confirm all disruptions	GCACGTCAAGACTGTCAAGG
NSO46	Insert *Xba*I 600 bp upstream of WSC1	AAAATCTAGAGCAAGACAGTTTACACAGCA
NSO47	Insert *Hin*dIII 400 bp downstream of WSC1	AAAAAAGCTTGCTATTAGTTTCATAACAAT
NSO75	Create Y303A mutation in WSC1	GGAAGCCCAAGAGGCGATA
NSO76	Create Y303A mutation in WSC1	CTCTTGGGCTTCCTTTTCCAT
NSO79	Create S19A-S20A mutation in WSC1	CGCCGCTGCATTTTCATCTA
NSO80	Create S19A-S20A mutation in WSC1	GAAAATGCAGCGGCGTATAGTT
NSO85	Create S22A-S23A mutation in WSC1	CATTTGCAGCTAATCACGGGCCCT
NSO86	Create S22A-S23A mutation in WSC1	GTGATTAGCTGCAAATGAAGAGGCGT
NSO88	Create L369A-V371A mutation in WSC1	CAACGTTGCAACAGCGGTCAATCCA
NSO89	Create L369A-V371A mutation in WSC1	GATTGACCGCTGTTGCAACGTTGTTT
NSO90	Create N373A-D375A mutation in WSC1	GTCGCTCCAGCCGAAGCTGAT
NSO91	Create N373A-D375A mutation in WSC1	CTTCGGCTGGAGCGACCGCT
NSO77	Create WSC1 cytoplasmic tail truncation mutant	CAGGATGGAACGGATCCCCGGGT
NSO78	Create WSC1 cytoplasmic tail truncation mutant	CGGGGATCCGTTCCATCCTGTCTT

### Overlay adhesion and immunofluorescence assays

The overlay adhesion assay was performed as described (Reynolds *et al.* 2008). Briefly, the biofilms were grown for approximately 5 days or more at 25° on 0.3% agar YPD plates and then a piece of plastic wrap (*i.e.*, Reynolds wrap) was placed over the biofilm and removed with both hands. This removed cells that were not agar-adherent.

Immunofluorescence of Flo11-HA^30^ on the cell surface of cells from the rim and hub of biofilms was performed as described by Reynolds *et al.* (2008). Briefly, after approximately 5 days of growth, small plugs of biofilm were taken using a pipette with the tip cut off and then were stained by secondary immunofluorescence using an anti-HA primary antibody.

### Western blotting

Precipitation of extracellular Flo11-HA^30,1015^p from the mat, fractionation of cells, and Western blotting were performed as described by Sarode *et al.* (2011).

### Site-directed mutagenesis

The mutagenesis was performed using a primer-mediated PCR-based method described in Fisher and Pei (1997) and Li and Mullins (2002) using primers listed in Table 2.

## Results

### Wsc1p affects mat formation in a Flo11p-independent manner

**Figure 1:**
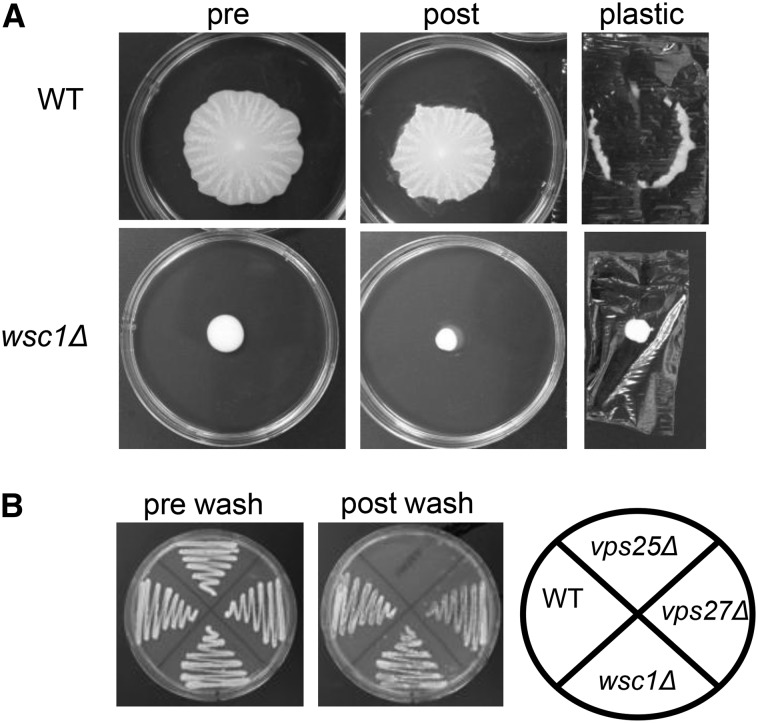
Wsc1p affects mat formation in a Flo11p-independent manner. (A) The overlay adhesion assay was performed on wild-type (WT) and *wsc1Δ* strains grown on low-agar plates. (B) The invasive growth assay was performed on WT, *wsc1Δ*, *vps25Δ* (Flo11p-dependent), and *vps27Δ* (Flo11p-independent) strains grown on solid 2% agar YPD plates.

Wsc1p is a sensor protein of the CWI pathway. It functions in parallel with other sensors (Mid2p, Wsc2p, and Wsc3p) to detect cell wall damage and activate the pathway (Figure S1). A defect in Wsc1p signaling can lead to increased sensitivity to cell wall–perturbing factors like high temperature, calcofluor white, and caffeine (Verna *et al.* 1997; Levin 2005; Straede and Heinisch 2007). As shown in Figure 1A, Wsc1p is also important for mat formation because *wsc1*∆ failed to form the typical patterned biofilm observed in the wild-type. It also adhered poorly in the overlay adhesion assay (Figure 1A). However, it displayed no defect for the Flo11p-dependent invasive growth phenotype (Figure 1B). Thus, its phenotypes were similar to the *vps27*∆ mutant (Sarode *et al.* 2011).

**Figure 2:**
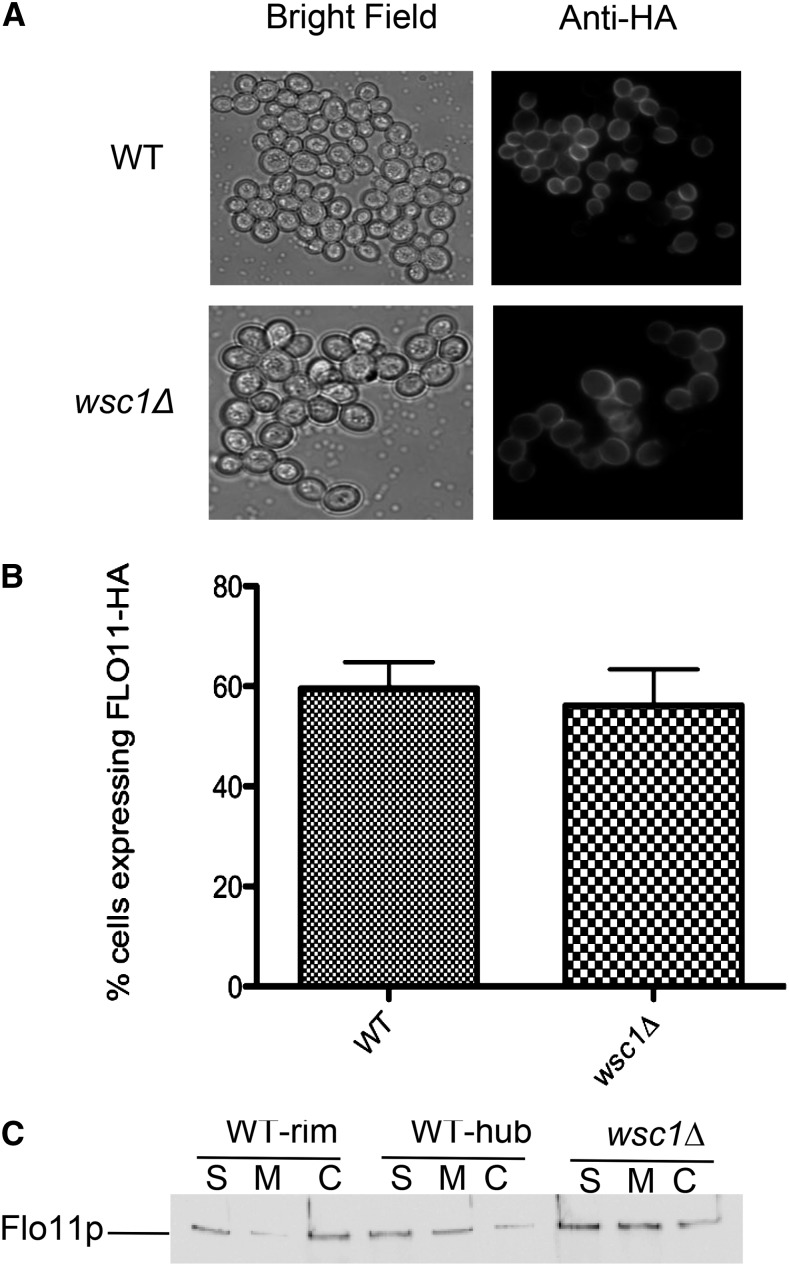
The *wsc1Δ* mutant shows no defect in Flo11-HA expression, localization, or shedding. (A) Cells were stained by secondary immunofluorescence with anti-HA antibody against Flo11-HA^30^. (B) A graph of the percentage of cells expressing Flo11-HA^30^ from each strain is shown. (C) Cell fractionation was performed on mats from wild-type and *wsc1∆* strains to separate shed (S), membrane-bound (M), and covalently attached (C) protein fractions, which were then analyzed by Western blotting using anti-HA monoclonal antibody to Flo11-HA^30,1015^.

To ascertain if the *wsc1*∆ mat formation defect was attributable to a defect in Flo11p expression or localization, the percentage of cells expressing Flo11p on the cell wall were measured by immunofluorescence. The strain carries an HA epitope tag inserted between residues 30 and 31 of Flo11p (Flo11-HA^30^) that can be stained using anti-HA antibody (Sarode *et al.* 2011). As can be seen in Figure 2A and Figure 2B, there was no statistically significant difference between wild-type and *wsc1*∆ in the number of cells expressing Flo11-HA^30^ on the cell surface.

In addition to being expressed on the cell wall surface, Flo11p also was recently reported to be shed outside the cell (Karunanithi *et al.* 2010; Sarode *et al.* 2011). To verify that *wsc1*∆ did not lead to any defects in Flo11p shedding, strains containing Flo11p tagged with an additional HA epitope tag at residue 1015 (Flo11-HA^30,1015^) were used. The mat cells were subjected to subcellular fractionation to separate populations of Flo11p that were shed extracellularly (S), covalently attached to the wall (C), and found in the membrane (M), and these were analyzed by SDS-PAGE and Western blotting using an anti-HA antibody (Figure 2C). Consistent with the immunofluorescence data, there appeared to be no reproducible difference in Flo11-HA^30,1015^ levels between wild-type and *wsc1*∆ strains.

One initial concern was that because the *wsc1∆* mutant has cell wall defects, it might have a slight growth defect that would correlate with its mat formation defect. However, the *wsc1∆* strain grew as well as wild-type (Figure S2); thus, its mat formation defects were not a consequence of growth defects.

### Cell wall integrity MAPK cascade is not essential for mat formation

Wsc1p is an important sensor of the CWI pathway (Figure S1), so we wanted to determine what downstream components of the pathway are required for mat formation. Deletion mutants of different components were analyzed for their effects on mat formation. Loss of other sensors of the Wsc family (*i.e.*, Wsc2p, Wsc3p) and Mid2p failed to cause any defect in mat formation, suggesting that Wsc1p is the major sensor of the CWI pathway for mat formation (data not shown). No mutants were generated for *PKC1* and *RHO1* because these genes are essential (Levin 2011).

**Figure 3:**
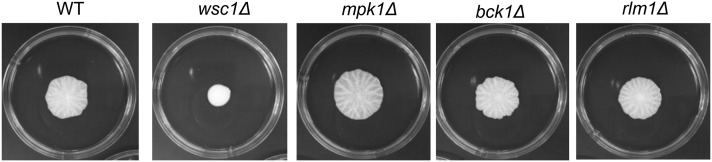
CWI–MAPK pathway components downstream of *PKC1* including the transcription factor Rlm1p are not necessary for mat formation. Mat formation phenotypes of wild-type (WT), *wsc1Δ*, MAP kinase cascade genes (bck1*Δ*, *mpk1Δ*), and downstream transcription factor *rlm1Δ* are shown after 5 days at 25°.

The CWI–MAPK cascade is one of the most well-characterized downstream effector pathways of Wsc1p, so it was examined first. Deletion mutants were generated for all nonredundant components of the CWI–MAPK cascade including *mpk1*∆, *bck1*∆, and the downstream transcription factor *rlm1*∆ (Levin 2011) (Figure S1). None of these mutations led to a defect in mat formation (Figure 3).

### Wsc1p–Rom2p interaction is essential for mat formation

**Figure 4:**
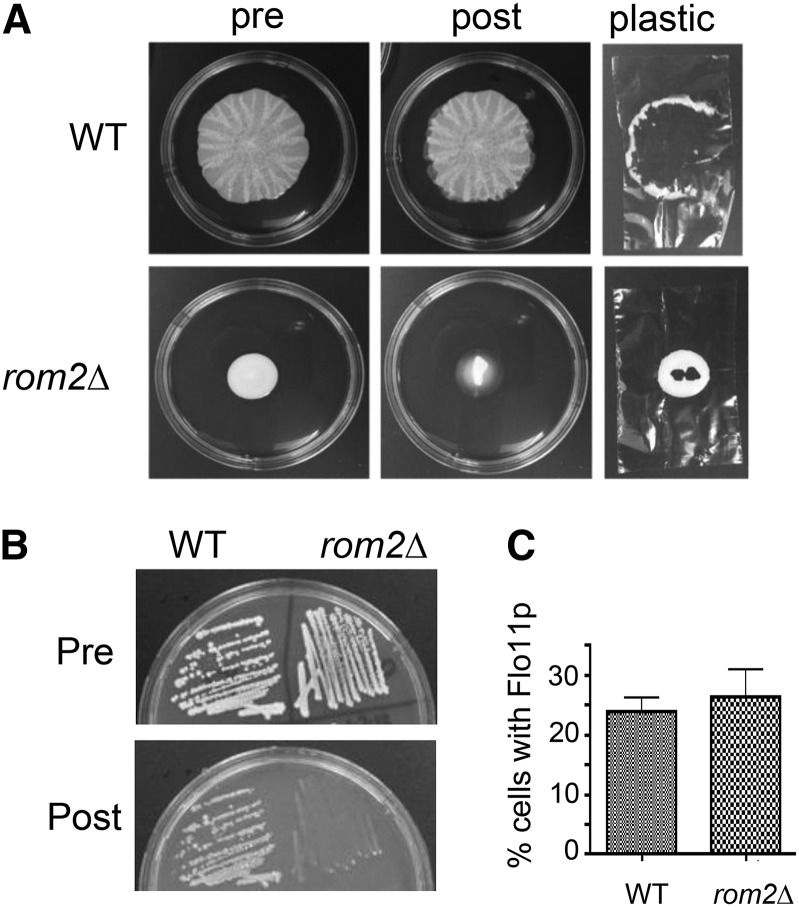
The *rom2Δ* mutant is defective in mat formation and invasive growth, but not Flo11p expression. (A) Overlay adhesion assay performed on wild-type (WT) and *rom2Δ*. (B) The invasive growth assay was performed on WT and *rom2∆* strains. (C) The percentage of cells expressing cell surface Flo11-HA^30,1015^ was calculated by immunofluorescent microscopy using an anti-HA primary antibody and then by comparing the fluorescing cells to the total number in a given field using bright-field optics.

The fact that the CWI–MAPK cascade is not involved in mat formation raised the question of which other canonical proteins are acting downstream of Wsc1p to affect mat formation. For example, the interaction between the sensor Wsc1p and the Rho1p GEF Rom2p is the primary step activating the CWI pathway (Philip and Levin 2001). Therefore, we tested to see if a *rom2Δ* mutant was compromised for mat formation, and it was, in fact, defective (Figure 4A). However, the *rom2∆* mutant differs from *wsc1∆* in that it exhibits defects in invasive growth (Figure 4B). The *rom2∆* mutant does not block expression of Flo11p on the surface of cells within the biofilm based on immunofluorescence analysis (Figure 4C). However, microscopic examination reveals a stark difference in the cellular distribution of Flo11p between the cells of the *rom2∆* and *wsc1∆* mutants. Cells collected from mats generated by wild-type and *wsc1∆* mutants are primarily found in clusters and the cells have stochastic expression of Flo11p, with a combination of fluorescing and nonfluorescing cells (Figure 2 and Figure S3). This is similar to what was seen in the *vps27∆* mutant (Sarode *et al.* 2011). In contrast, the *rom2∆* mutant has a large number of single cells and few of these cells express Flo11p. It has small clusters of cells as well; in these clusters, Flo11p expression is very clear (Figure S3). The wild-type, *vps27∆*, and *wsc1∆* mutants exhibit very few single cells in comparison (Figure 2) (Sarode *et al.* 2011).

Wsc1p is known to act through Rom2p, but the *rom2∆* mutant may have a stronger defect because Rom2p interacts with both Mid2p and Wsc1p to transduce signals downstream (Philip and Levin 2001). When both of these proteins are disrupted, the cell wall suffers additive defects. Thus, a disruption of Rom2p will result in greater cell wall damage than the *wsc1∆* mutation alone, which may explain the additional defects to invasive growth observed in the *rom2∆* mutant. In addition, Rom2p may impact many other pathways through Rho1p, causing it to be more pleiotropic (see *Discussion*).

To better-elucidate whether Wsc1p acts through Rom2p *vs.* some other signaling module to affect mat formation specifically, given the pleotropic effect of a complete *rom2∆* disruption, residues in the Wsc1p cytoplasmic tail that are known to interact with Rom2p were mutated to determine if interruption of these protein–protein interactions would compromise mat formation in a specific manner. Vay *et al.* (2004) used mutational analysis of the cytoplasmic tail of Wsc1p to identify the residues important for Wsc1p–Rom2p interactions. They determined residues Y303, S319-320, S322-323, L369, V371, N373, and D375 to be crucial. The Y303 and L369-D375 residues were particularly important. The S319-323A mutations could actually suppress some of the other mutations. If mutations that block the Wsc1p–Rom2p interaction also block mat formation, then this will support that Wsc1p–Rom2p interactions are important for mat formation. Because Rom2p is a well-known activator of Rho1p (Ozaki *et al.* 1996), this will strongly implicate Rho1p and establish a role for the primary upstream interaction of Wsc1p with Rom2p in the CWI pathway (Figure S1).

The fusion gene of *WSC1* regulated by the *WSC1* promoter was subcloned into a vector such that it encoded Wsc1p with a green fluorescent protein (GFP) tag on the C-terminal cytoplasmic tail. Transformation of the *wsc1*∆ mutant with *WSC1-GFP* (*wsc1*∆::*WSC1-GFP*) led to rescue of mat formation (Figure S4A) and temperature sensitivity phenotypes (Figure S4B), thus confirming that it is fully functional .

The aforementioned amino acids that mediate Wsc1p–Rom2p interactions were mutated to alanine. Constructs were generated that carried a single-point mutation (Y303A), double-point mutations (S319A S320A or L369A V371A), and triple-point mutations (Y303A L369A V371A). No transformants could be obtained for point mutations in the C-terminal–most region of the cytoplasmic tail (N373, D375) either by themselves or in combination with any other point mutations, and the reason for this is unknown.

**Figure 5:**
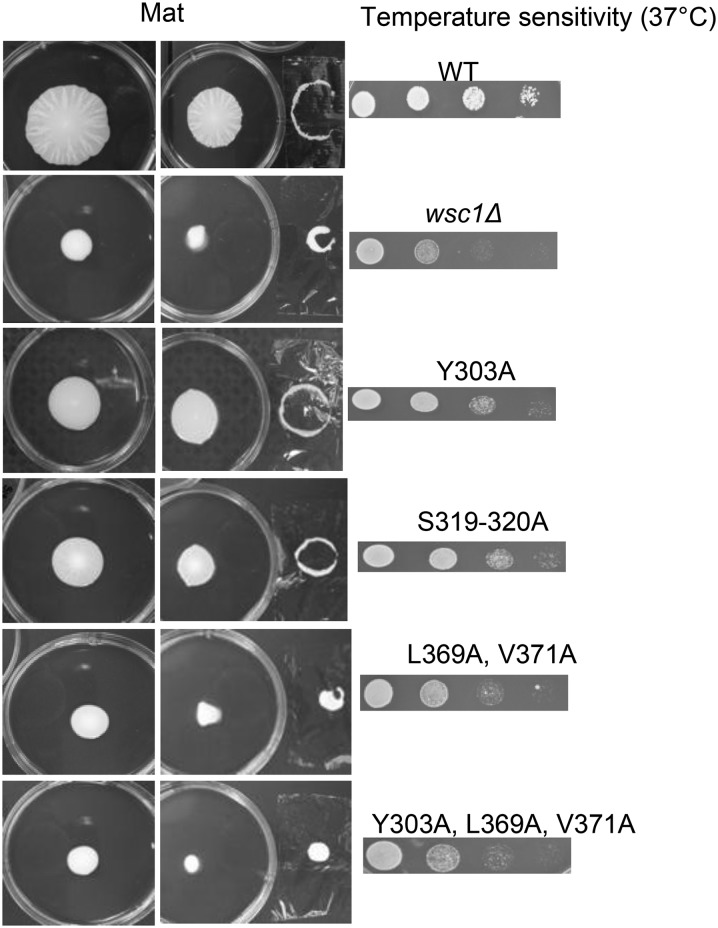
Wsc1p–Rom2p interaction is essential for mat formation. The overlay adhesion assay was performed on mats formed by *WSC1-GFP* point mutants, along with corresponding temperature sensitivity assays of every mutant.

None of the point mutants fully complemented the mat formation or temperature-sensitive growth defects (Figure 5); however, the Y303A and S319A S320A mutants did complement both phenotypes better than the L369A V371A, or Y303A L369A V371A mutants. Thus, both mutant phenotypes appeared to increase in severity as the location of the mutations edged closer to the extreme C-terminus. In contrast to observations of Vay *et al.* (2004), we did not observe any growth defect at 30° in any of our mutants. This could be attributable to the fact that they performed the mutations and complementation study in a *wsc1∆ mid2∆* double-mutant that exhibited a severe lysis defect at all growth temperatures in absence of osmotic support.

**Figure 6:**
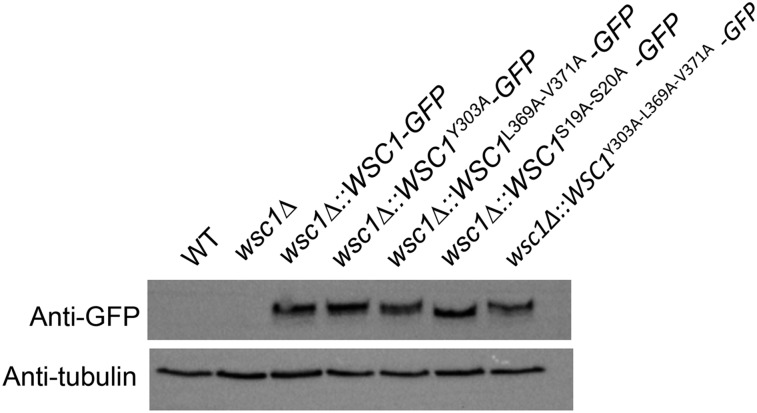
Wsc1p-GFP mutant proteins are expressed similar to those of wild-type. Strains were grown in liquid YPD to log phase. Lysates were generated and then probed for GFP with tubulin as a loading control.

One concern was that the failure of the Wsc1p mutations to fully complement the *wsc1∆* phenotypes was attributable to poor expression of the mutant proteins. However, the mutated versions of Wsc1-GFP were expressed similarly to the wild-type protein (Figure 6). Some of the Wsc1p mutations did cause a mild decrease in growth rate of the strain compared to wild-type, the Wsc1p-GFP strain, or even the *wsc1∆* mutant itself (Figure S2). However, this slower growth rate does not explain the mat formation defect because an *itr1∆ itr2∆* mutant, which also has a similar mild growth defect, still generates a mat similar to the wild-type but at a slightly slower rate (Figure S2). In contrast, the Wsc1-GFP mutants that failed to complement or only partially complemented, grew as well in liquid media as *itr1∆ itr2∆* but still formed a defective mat. The *wsc1∆*::*WSC1*^Y303A-L369A-V371A^*-GFP* mutant grew even more slowly than the others, but the *wsc1∆*::*WSC1^L369A-V371A^-GFP* had a mat phenotype similar to this mutant and a growth rate similar to the *itr1∆ itr2∆* mutant. Thus, growth rate clearly is not a major factor.

### Role of Skn7 in mat formation

**Figure 7:**
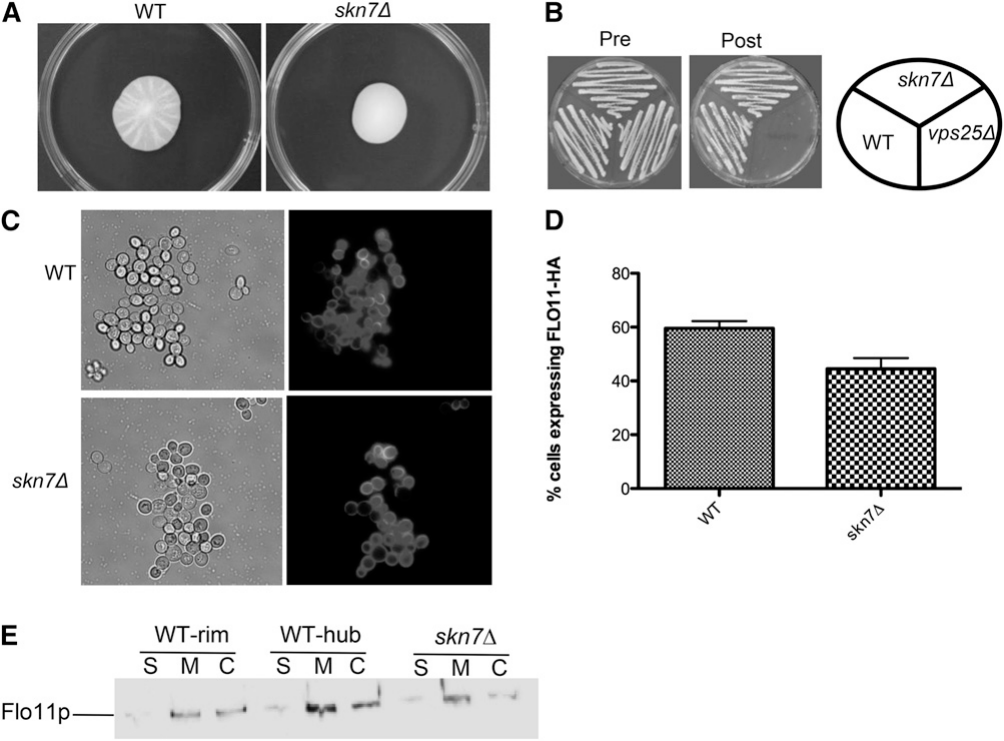
Skn7p affects mat formation in a Flo11p-independent manner. (A) Mat formation and (B) invasive growth assay were performed on wild-type (WT) and *skn7Δ* strains. (C) The level of Flo11-HA^30^ at the cell surface was measured using secondary immunofluorescence with an anti-HA monoclonal antibody and the results expressed as the percentage of cells expressing Flo11-HA^30^ from each strain are graphed in (D). (E) A Western blot of shed (S), membrane-bound (M), and covalently attached (C) protein fractions from each strain isolated from the rim or hub or whole mat (in the case of *skn7∆*) were analyzed by Western blotting with a primary anti-HA monoclonal antibody.

The fact that the Wsc1p–Rom2p interaction is needed for mat formation, but the CWI–MAPK cascade is not suggests that the biofilm pathway might be mediated via Rho1p through another downstream effector. One option is the Skn7p transcription factor that acts downstream of Rho1p and parallel to the Pkc1p branch of the CWI pathway (Alberts *et al.* 1998; Levin 2011) (Figure S1). Therefore, a *skn7∆* mutant was generated and tested. This mutant is defective in mat formation (Figure 7A). However, the mutant shows no defect in invasive growth, Flo11p localization, or expression and shedding of Flo11p, based on the invasive growth assay (Figure 7B), Flo11-HA^30,1015^ immunofluorescence (Figure 7, C and D), and Western blotting (Figure 7E) assays, respectively. Thus, like the *wsc1∆* mutant, the *skn7Δ* mutant is defective in mat formation in a Flo11p-independent manner.

**Figure 8:**
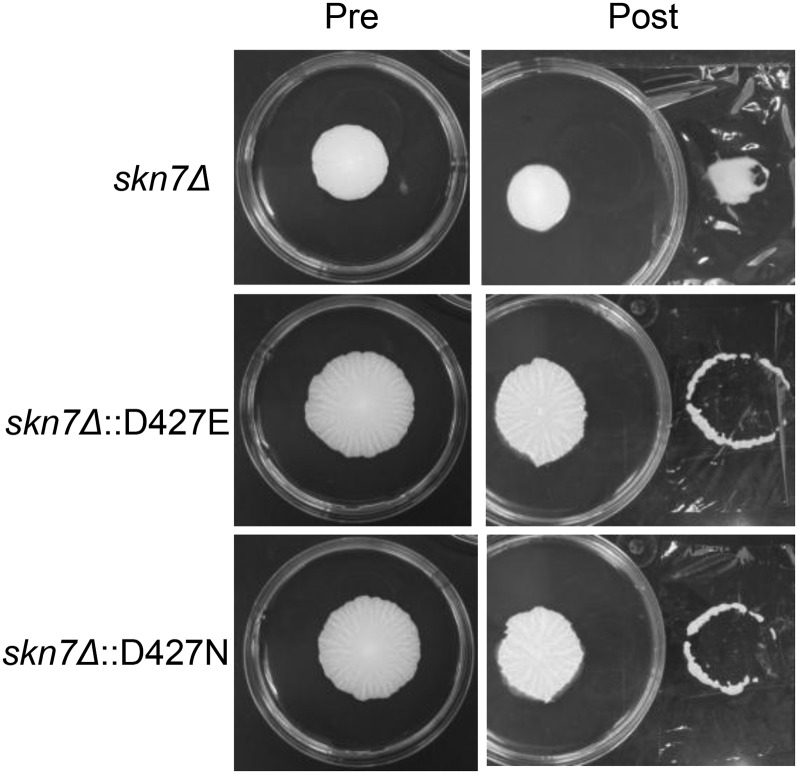
Sln1p-Skn7p branch is not essential for mat formation. Mat formation and overlay adhesion assays were performed on the Skn7^D427^ point mutants.

In addition to functioning downstream of Rho1p in the CWI pathway, Skn7 has other distinct roles within the cell, including oxidative stress response regulation, and it also acts downstream of the Sln1p histidine kinase as a response regulator to affect the cell wall (Ketela *et al.* 1998; Li *et al.* 1998). A conserved aspartic acid residue at position 427 in the receiver domain of Skn7p is known to be essential for its function in the Sln1p-dependent pathway (Brown *et al.* 1993, 1994; Ketela *et al.* 1998; Li *et al.* 1998). Mutating the aspartic acid to glutamic acid (D427E) generated a hyperactive form of Skn7p, whereas a mutation to asparagine (D427N) diminished its activity (Li *et al.* 1998). Plasmids carrying *SKN7* genes with point mutations pCLM699 (Skn7D427N) and pCLM700 (Skn7D427E) (Li *et al.* 1998) were transformed into the *skn7∆* mutant. If phosphorylation of the Skn7p conserved aspartic acid residue on the receiver domain plays a role in mat formation, then the hyperactive version of Skn7p (D427E) should rescue the mat formation defect of *skn7*∆, whereas the inactive version (D427N) should fail to do so. Complementing the *skn7*∆ mutation with both the active and inactive mutant forms of Skn7p led to rescue of the mat formation defect of *skn7*∆ (Figure 8). This suggests that Skn7p is not acting downstream of Sln1p to control mat formation, but rather is acting downstream of Rho1p. Thus, its activities in mat formation must be mediated by a D427-independent mechanism (Alberts *et al.* 1998; He *et al.* 2009), which is likely to be through the Wsc1p-Rom2p-Rho1p pathway.

## Discussion

It was shown that components of the CWI pathway are required for mat formation in a manner that is independent of Flo11p and the canonical CWI–MAPK cascade. The involvement of the CWI pathway in mat formation begins with Wsc1p, which is a sensory protein of the CWI pathway (Figure S1), whose activation has diverse effects on the cell wall (Raymond *et al.* 1992; Piao *et al.* 2007; Hurley 2008).

Our data indicate that the Wsc1p–Rom2p interaction is essential for mat formation. The *rom2∆* and *wsc1∆* mutants share overlapping phenotypes, having defects in mat formation but still expressing Flo11p (Figure 1, Figure 2, and Figure 4). The caveat to these data is that the *rom2∆* mutant has more cell–cell association defects than *wsc1∆* and, unlike the sensor mutant, it is defective for invasive growth (Figure 4). However, Rom2p receives signals from a number of additional cell wall sensors such as Mid2p (Philip and Levin 2001), so it is possible that its increased cell wall defects could translate into defects in invasive growth in addition to mat formation. Moreover, Rho1p regulates a number of other effectors, including Bni1p (actin), Fks1p-Fks2p (β1,3-glucan synthase), and the CWI–MAPK cascade (Levin 2005). Thus, the defects may be greater in the *rom2∆* mutant because it impacts multiple pathways, whereas *wsc1∆* only impacts a subset of pathways (Popolo and Vai 1999).

However, the importance of the Wsc1p–Rom2p interaction for mat formation was also supported by site-directed mutagenesis studies showing that mutating the Wsc1p cytoplasmic tail residues necessary for interaction with Rom2p leads to defects in mat formation (Figure 5). The results for the L369A and Y371A mutations are consistent with those of Vay *et al.* (2004) in that mutations in these C-terminal amino acids completely blocked the function of Wsc1p. The Y303A mutant was also defective for mat formation, although not as defective as the L369A Y371A mutant. This contrasts slightly with the results of Vay *et al.* (2004), who found that the phenotypes for Y303A and L369A Y371A mutations in growth were more similar. The reasons for this contrast in the studies are unclear, and these differences may also help to explain why the effects of S319-320A mutations on mat formation are more difficult to compare to those of Vay *et al.* (2004) for growth. S319-320A mutations, when expressed as the sole alterations to Wsc1p, caused a defect in mat formation that was not a severe as the L369A V371A mutant (Figure 5). In contrast, Vay *et al.* (2004) found that S319-320A mutations could slightly suppress the growth defect of a Wsc1p mutant protein carrying a deletion of the Wsc1p C-terminus including the L369-D375 amino acids. These studies are difficult to compare for several reasons. First, the strain backgrounds differ. Second, their assays were all performed in a strain lacking both *wsc1∆* and *mid2∆*, and therefore the strain had a more severe cell wall defect. Given the differences in the studies, the overlap we found between their results and ours regarding amino acids that affected Wsc1p–Rom2p interactions support the model that Wsc1p acts through Rom2p to control mat formation.

A surprising result from these studies is that the CWI–MAPK cascade is not involved in mat formation (Figure 3). This suggests that Wsc1p–Rom2p acts through another downstream pathway; based on the Wsc1p–Rom2p interactions, we suggest that this unknown pathway likely branches out from the GTPase Rho1p, which is regulated by Rom2p (Levin 2005). Rho1p is an essential GTPase at the center of a regulatory network that has effectors that control cell wall biogenesis through polarization of actin cytoskeleton, activation of the transcription factor Skn7p, and β-glucan synthesis (Levin 2005) (Figure S1).

Of these possibilities, mutant analysis implicates Skn7p. A *skn7Δ* mutant, like *wsc1Δ*, is defective in mat formation in a Flo11-dependent manner, but not invasive growth. The other possibilities are less likely, although not completely ruled out, based on the following logic. A *bni1∆* mutant that represents the Bni1p protein that acts downstream of Pkc1p independently of the CWI–MAPK cascade to affect the actin cytoskeleton is defective for mat formation (Evangelista *et al.* 1997) but also has defects in invasive growth (data not shown). This is similar with the phenotype of Rom2p and may be related to the rom2Δ mutant's phenotype. The *fks1∆* and *fks2∆* mutants did not have any defects in mat formation, and a double mutant is unviable (Mazur *et al*. 1995). However, it has been shown that disruption of β1,3-glucan perturbs invasive growth (Birkaya *et al.* 2009), and we did not see this in the *wsc1∆* or *skn7∆* mutants (Figure 1 and Figure 8) Thus, the phenotypes of the *skn7∆* mutant are most like the *wsc1∆* mutant, suggesting that Skn7p plays a role downstream of Wsc1p in mat formation.

However, Skn7p is regulated by both the CWI pathway and high-osmolarity glycerol (HOG) signaling pathways (Figure S1). The genes activated by Skn7p, as a consequence of its activation through the HOG pathway via Sln1p, are not identical to those activated through the CWI pathway (Ketela *et al.* 1998; Li *et al.* 1998). This is because Skn7p is a modular transcription factor that can affect different sets of genes through distinct domains, depending on which pathway activates it (Alberts *et al.* 1998; He *et al.* 2009).

The Sln1p histidine kinase activates Skn7p by phosphorylating the D427 residue, resulting in upregulation of certain target genes, including *OCH1*, which encodes a glycosyltransferase in the Golgi complex (Li *et al.* 2002). To determine if Skn7p causes a defect in mat formation downstream of Sln1p, hyperactive (D427E) or inactive (D427N) point mutants of *SKN7* that either over-respond or under-respond to the Sln1p branch of the HOG pathway, respectively, were transformed into *skn7Δ*. Because both point mutants rescued the mat formation phenotype, it was shown that Skn7p does not act downstream of Sln1p to affect mat formation.

Taking the data altogether, we propose a model suggesting that Wsc1p regulates mat formation by affecting the activation of the Skn7p transcription factor through the Rom2p-Rho1p module. This model is not completely proven by these data, and it is possible that other factors such as Bni1p or another unknown factor are involved, but our data suggest this model, which will be tested in the future.

In addition, we recently showed that one Flo11p-independent mat formation pathway, referred to as the biofilm pathway, involves the class E vacuolar protein sorting (vps) components of the MVB pathway. It was hypothesized in our previous work (Sarode *et al.* 2011) that the biofilm pathway would involve a cell wall sensory protein whose mislocalization in class E vps mutants results in defective mat formation. It is possible that Wsc1p is this protein, and components of the CWI pathway, including Wsc1p, Rom2p, Rho1p, and Skn7p, could be part of the Flo11p-independent biofilm pathway. Wsc1p localization depends on its recycling through a properly functioning endosomal MVB pathway, and a *vps27∆* mutant, which disrupts MVB sorting, traps Wsc1p in an aberrant endosome known as the class E compartment (data not shown) (Piao *et al.* 2007). The *wsc1∆* and *skn7∆* mutants share similar phenotypes with *vps27∆* by affecting mat formation, but not invasive growth or Flo11p expression and localization. Thus, Wsc1p may be at the head of a biofilm pathway affected by Vps27p, but this has yet to be solidly supported. Attempts to show this by epistasis have not been successful because of technical difficulties in overexpressing the components of the Wsc1p-Rom2p-Rho1p pathway in the ∑1278b strain background (data not shown). A future goal will be to elaborate on this pathway.

### Biofilm and CWI pathways have differential effects on mat formation in different ∑1278b strains

We have found that the CWI pathway affects mat formation in a manner that is independent of the CWI–MAPK cascade and may be affected by endosomal sorting mutations. However, Birkaya *et al.* (2009) recently found that in another ∑1278b strain, PC538, the CWI–MAPK cascade affected mat formation, invasive growth, and *FLO11* expression, and these were different from our findings. In addition to the differences in the way the CWI–MAPK cascade affects mat formation, the PC538 strain also differs from TRY181 in expressing *FLO10* (Birkaya *et al.* 2009), having much more wrinkled mats and being less affected by mutations in *VPS27* and other vps mutants (data not shown).

These phenotypic inconsistencies between the strains may be attributable to undefined genetic differences in PC538 and TRY181 (derived from L6906). One possible difference that could contribute is the fact that PC538 carries a *ste4∆* mutation, which could affect other signaling pathways. However, there may be other differences as well. Unraveling the differences between these strains will be valuable in understanding how mat formation is regulated in yeast.

## Supplementary Material

Supporting Information
