# Supplementary material for: The Wsc1p Cell Wall Signaling Protein Controls Biofilm (Mat) Formation Independently of Flo11p in Saccharomyces cerevisiae
Source: G3 (Bethesda). 2013 Dec 6;4(2):199–207. doi: 10.1534/g3.113.006361 (PMC3931555; doi:10.1534/g3.113.006361)
Supplement: Supporting Information [file supp_4_2_199__index.html]

The Wsc1p Cell Wall Signaling Protein Controls Biofilm (Mat) Formation Independently of Flo11p in Saccharomyces cerevisiae — Supporting Information 

# The Wsc1p Cell Wall Signaling Protein Controls Biofilm (Mat) Formation Independently of Flo11p in *Saccharomyces cerevisiae*

## Supporting Information for Sarode *et al.*, 2014

**Files in this Data Supplement:**

- Supporting Information - Figures S1-S4 (PDF, 1 MB)
- Figure S1 - Model depicting CWI and Sln1p cell wall signaling pathways. (PDF, 494 KB)
- Figure S2 - The growth rates of the *WSC1-GFP* strains do not impact mat formation. (PDF, 730 KB)
- Figure S3 - The *rom2Δ* mutant has fewer aggregated cells than the wild-type, and most of these cells do not express Flo11p. (PDF, 493 KB)
- Figure S4 - WSC1-GFP construct rescues mat formation defect and temperature sensitivity phenotypes of *wsc1Δ*. (PDF, 372 KB)
