# Supplementary material for: The Wsc1p Cell Wall Signaling Protein Controls Biofilm (Mat) Formation Independently of Flo11p in Saccharomyces cerevisiae
Source: G3 (Bethesda). 2013 Dec 6;4(2):199–207. doi: 10.1534/g3.113.006361 (PMC3931555; doi:10.1534/g3.113.006361)
Supplement: Supporting Information [file supp_g3.113.006361_006361SI.pdf]

G3 Genes | Genomes | Genetics

The Wsc1p cell wall signaling protein controls biofilm (mat) formation independently of Flo11p in *Saccharomyces cerevisiae*

Neha Sarode[1], Sarah E. Davis, Robert N. Tams, and Todd B. Reynolds*

Department of Microbiology, University of Tennessee, Knoxville, TN 37996

[1]Current address for Dr. Sarode: Department of Biology, Georgia Institute of Technology, Atlanta, GA 30332

*Corresponding author
Todd B. Reynolds, Associate Professor
University of Tennessee
Department of Microbiology
F321 Walters Life Sciences Bldg.
Knoxville, TN 37996
Ph: 865-974-4025
Email: treynol6@utk.edu

DOI: 10.1534/g3.113.006361

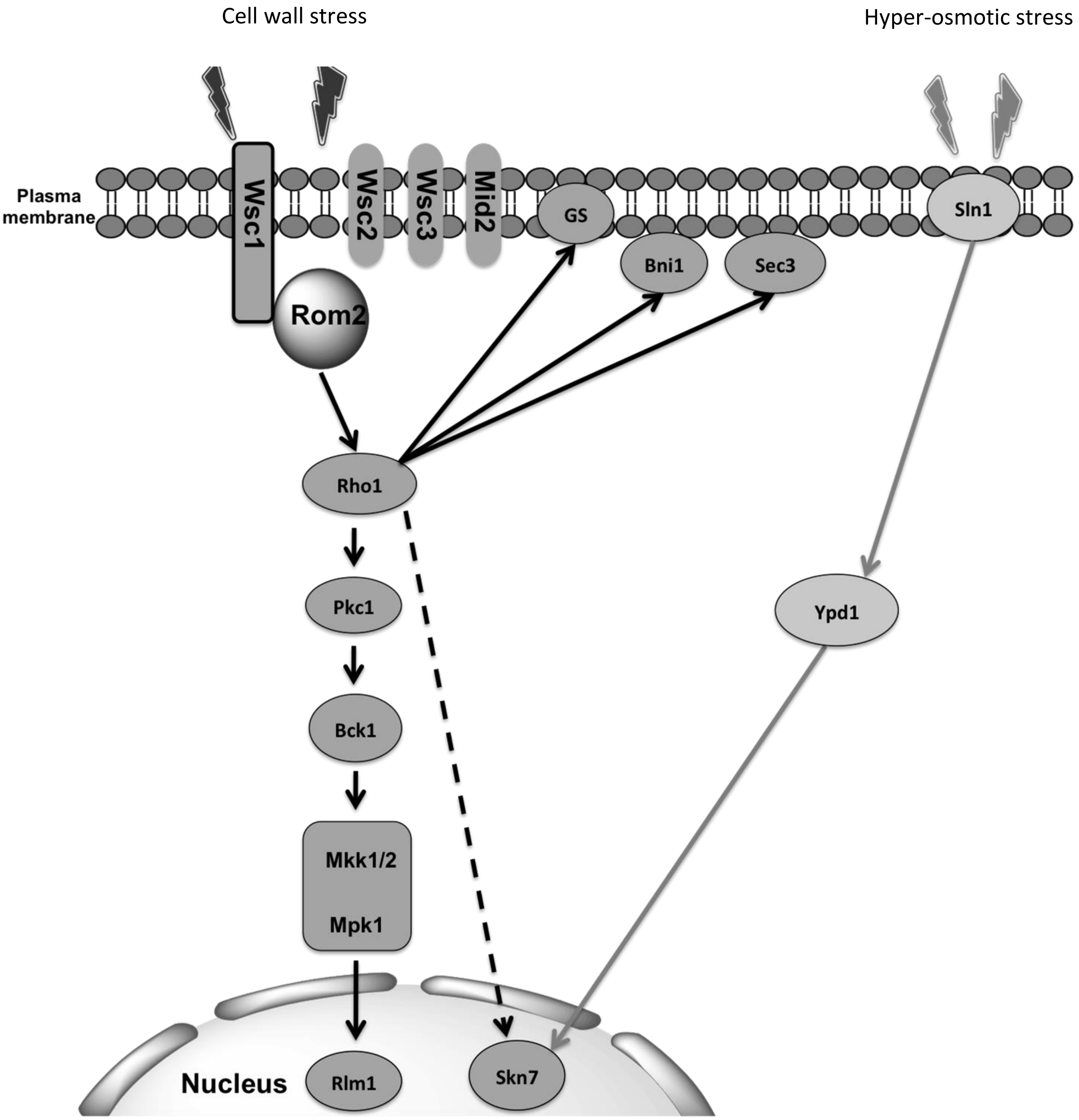

Figure S1 Model depicting CWI and Sln1p cell wall signaling pathways. The CWI pathway consists of cell wall stress sensors (WSC family members along with Mid2p) acting upstream of the small G-protein Rho1p and its GTP Exchange Factor (GEF) Rom2p. Among the effectors of Rho1p that regulate the cell wall is Pkc1p, which regulates a MAPK cascade (Bck1p, Mkk1/2p, and Mpk1p) that ultimately activates the cell wall stress response transcription factor Rlm1p. In addition, Rho1p regulates the cell wall transcription factor Skn7p. However, Skn7p also acts downstream of the Sln1p histdine kinase to affect the cell wall.

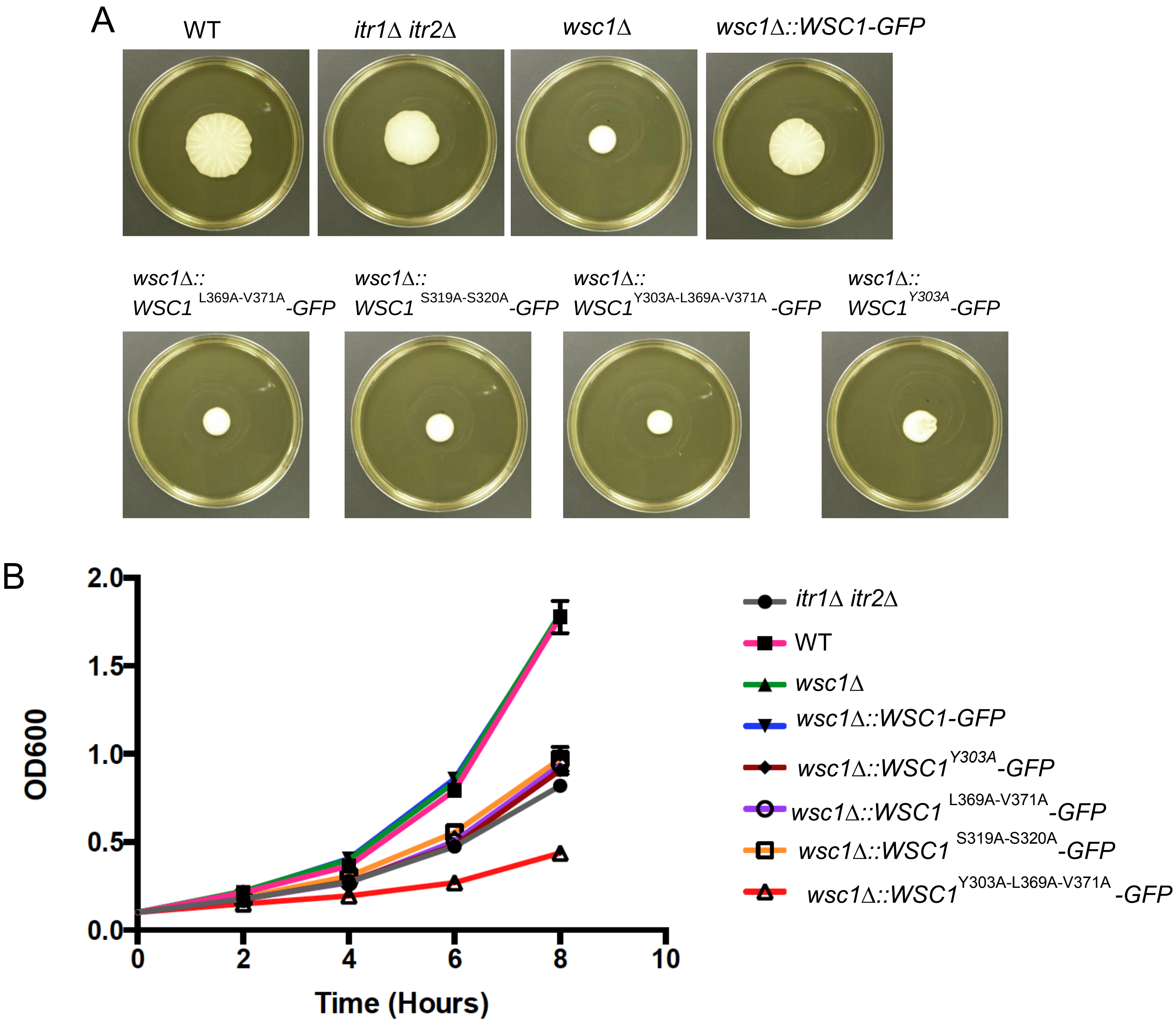

Figure S2 The growth rates of the *WSC1-GFP* strains do not impact mat formation. (A) Wild-type (WT) and *itr1Δ itr2Δ* mutants, that exhibit differing growth rates were compared for mat formation along with the *wsc1Δ* mutant and various *WSC1* point mutants. (B) The growth rate of the strains was compared in liquid media.

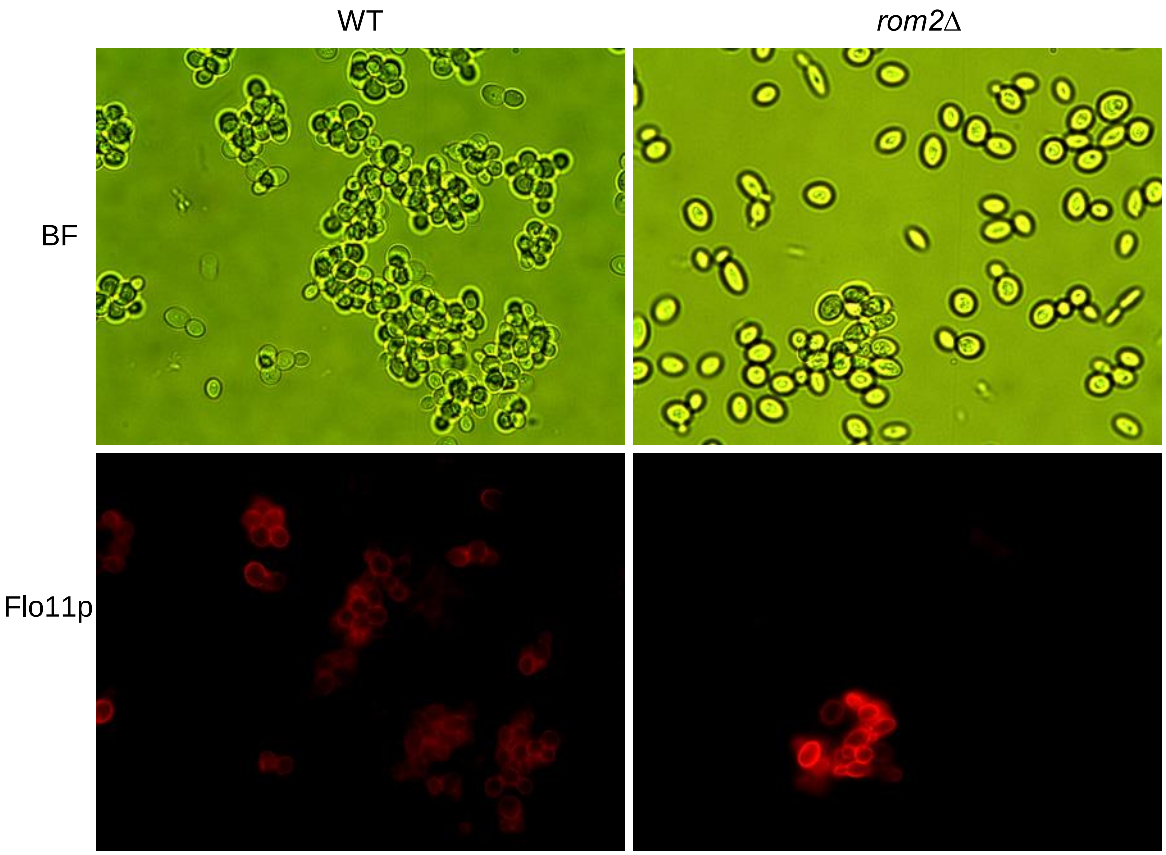

Figure S3 The *rom2Δ* mutant has fewer aggregated cells than the wild-type, and most of these cells do not express Flo11p. Cells from WT and *rom2Δ* were compared by immunofluorescent microscopy with an anti-HA antibody.

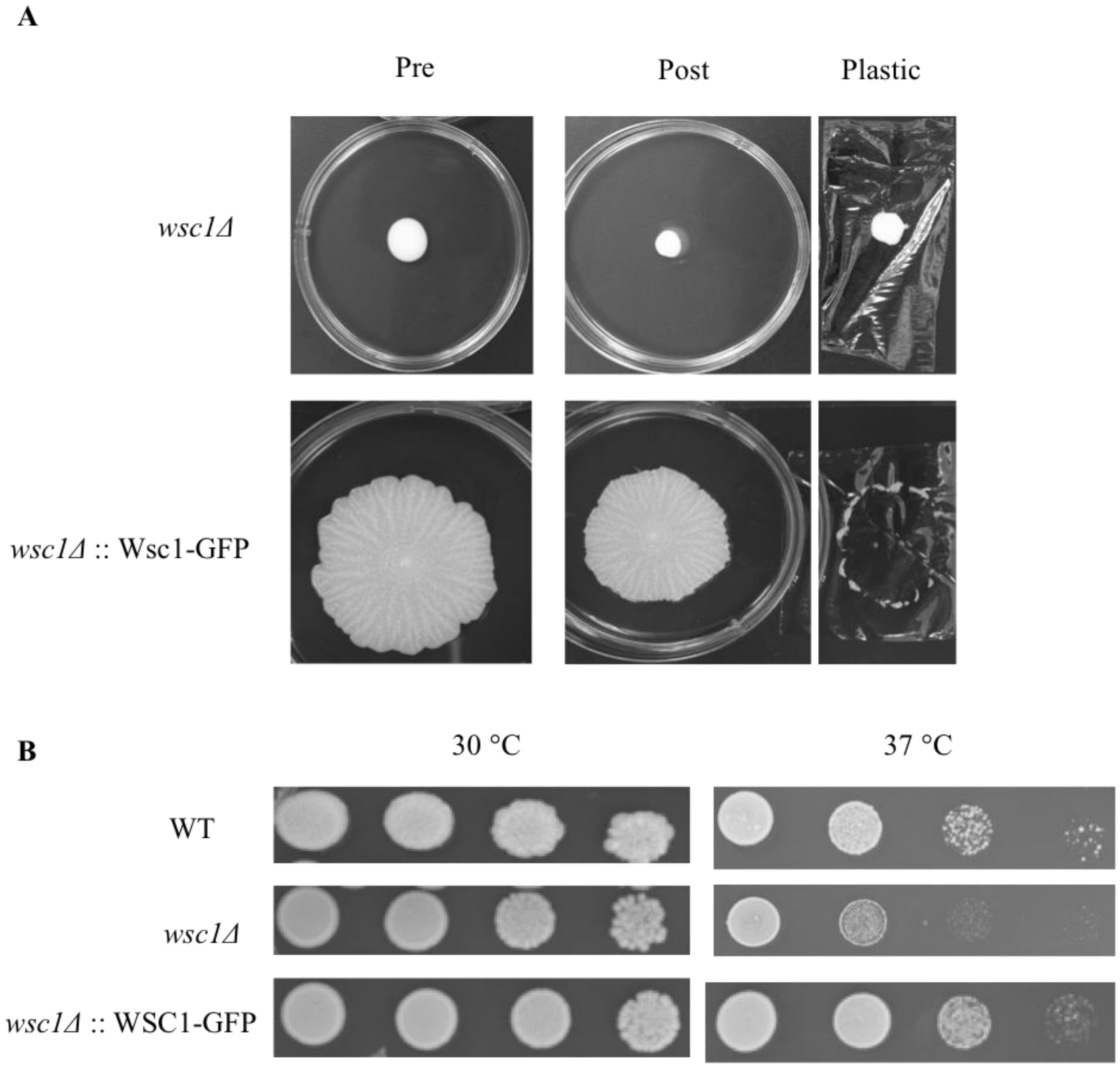

Figure S4 WSC1-GFP construct rescues mat formation defect and temperature sensitivity phenotypes of wsc1*Δ.*
(A) The mat formation phenotype and behavior in the overlay adhesion assay was assessed for the wild-type, *wsc1Δ,* and *wsc1Δ::WSC1-GFP* strains. (B) Growth of the strains was assessed after 48hrs at 37˚C.
