# Supplementary material for: The Wsc1p Cell Wall Signaling Protein Controls Biofilm (Mat) Formation Independently of Flo11p in Saccharomyces cerevisiae
Source: G3 (Bethesda). 2013 Dec 6;4(2):199–207. doi: 10.1534/g3.113.006361 (PMC3931555; doi:10.1534/g3.113.006361)
Supplement: Supporting Information [file supp_g3.113.006361_FigureS1.pdf]

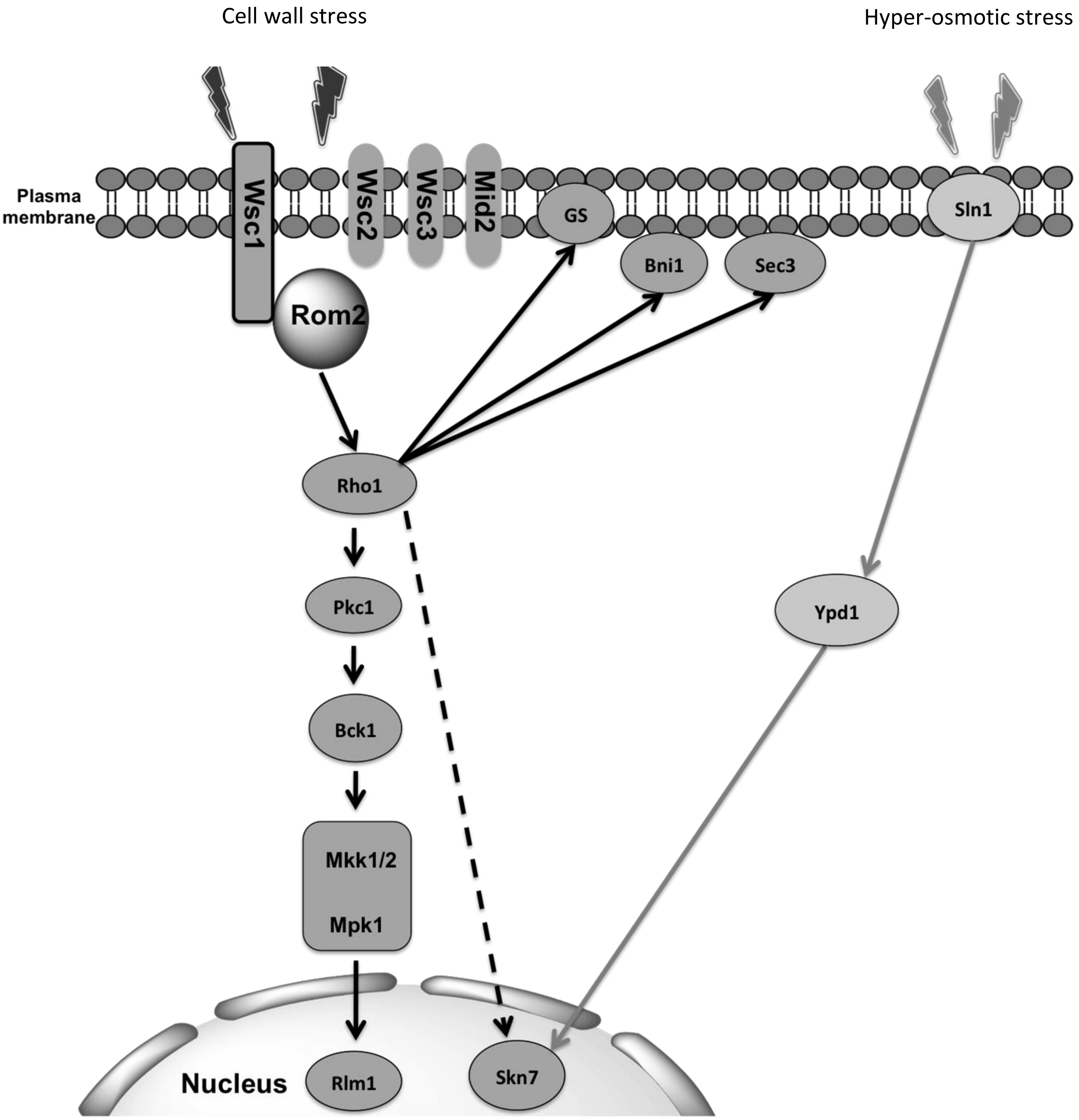

Figure S1 Model depicting CWI and Sln1p cell wall signaling pathways. The CWI pathway consists of cell wall stress sensors (WSC family members along with Mid2p) acting upstream of the small G-protein Rho1p and its GTP Exchange Factor (GEF) Rom2p. Among the effectors of Rho1p that regulate the cell wall is Pkc1p, which regulates a MAPK cascade (Bck1p, Mkk1/2p, and Mpk1p) that ultimately activates the cell wall stress response transcription factor Rlm1p. In addition, Rho1p regulates the cell wall transcription factor Skn7p. However, Skn7p also acts downstream of the Sln1p histdine kinase to affect the cell wall.
