# Supplementary material for: The Wsc1p Cell Wall Signaling Protein Controls Biofilm (Mat) Formation Independently of Flo11p in Saccharomyces cerevisiae
Source: G3 (Bethesda). 2013 Dec 6;4(2):199–207. doi: 10.1534/g3.113.006361 (PMC3931555; doi:10.1534/g3.113.006361)
Supplement: Supporting Information [file supp_g3.113.006361_FigureS2.pdf]

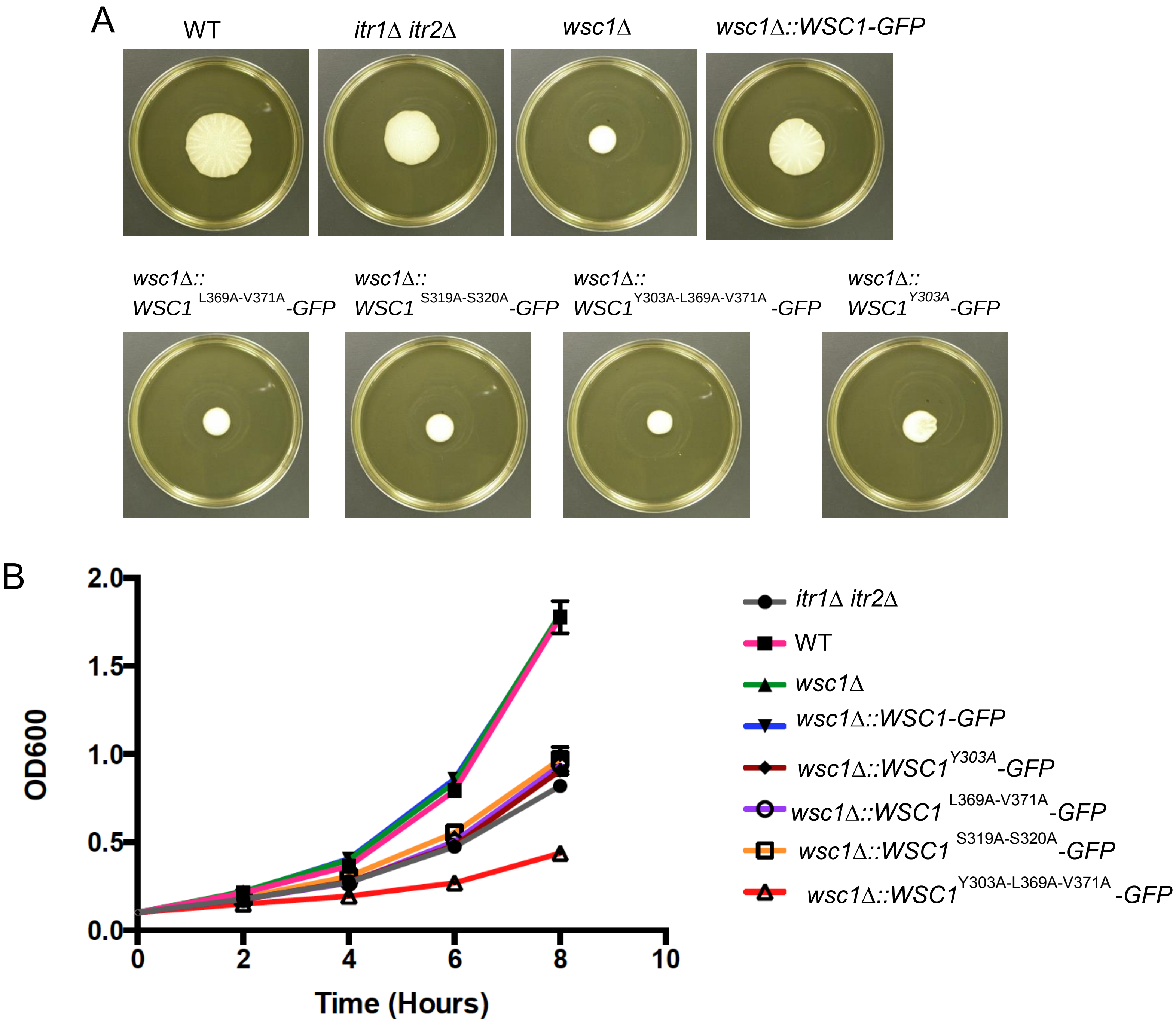

Figure S2 The growth rates of the *WSC1-GFP* strains do not impact mat formation. (A) Wild-type (WT) and *itr1Δ itr2Δ* mutants, that exhibit differing growth rates were compared for mat formation along with the *wsc1Δ* mutant and various *WSC1* point mutants. (B) The growth rate of the strains was compared in liquid media.
