# Supplementary material for: The Wsc1p Cell Wall Signaling Protein Controls Biofilm (Mat) Formation Independently of Flo11p in Saccharomyces cerevisiae
Source: G3 (Bethesda). 2013 Dec 6;4(2):199–207. doi: 10.1534/g3.113.006361 (PMC3931555; doi:10.1534/g3.113.006361)
Supplement: Supporting Information [file supp_g3.113.006361_FigureS3.pdf]

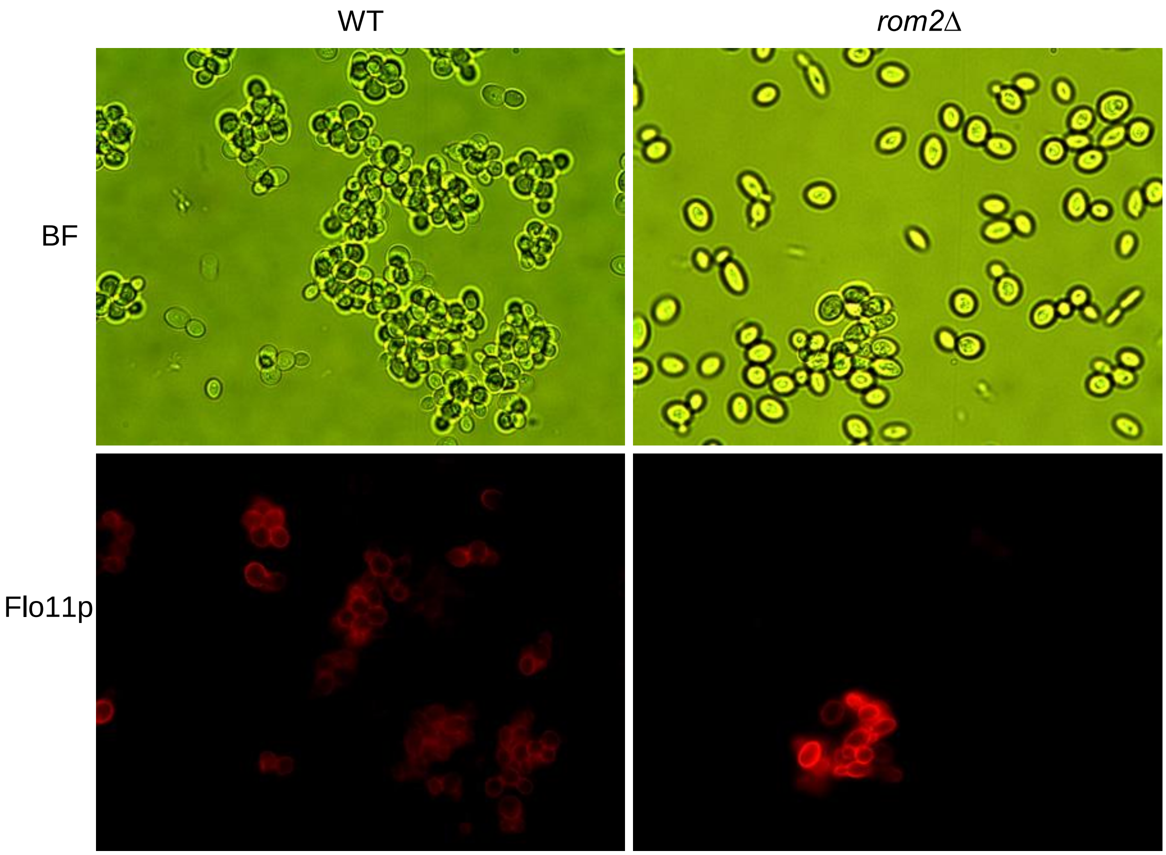

Figure S3 The *rom2Δ* mutant has fewer aggregated cells than the wild-type, and most of these cells do not express Flo11p. Cells from WT and *rom2Δ* were compared by immunofluorescent microscopy with an anti-HA antibody.
