# Supplementary material for: The Wsc1p Cell Wall Signaling Protein Controls Biofilm (Mat) Formation Independently of Flo11p in Saccharomyces cerevisiae
Source: G3 (Bethesda). 2013 Dec 6;4(2):199–207. doi: 10.1534/g3.113.006361 (PMC3931555; doi:10.1534/g3.113.006361)
Supplement: Supporting Information [file supp_g3.113.006361_FigureS4.pdf]

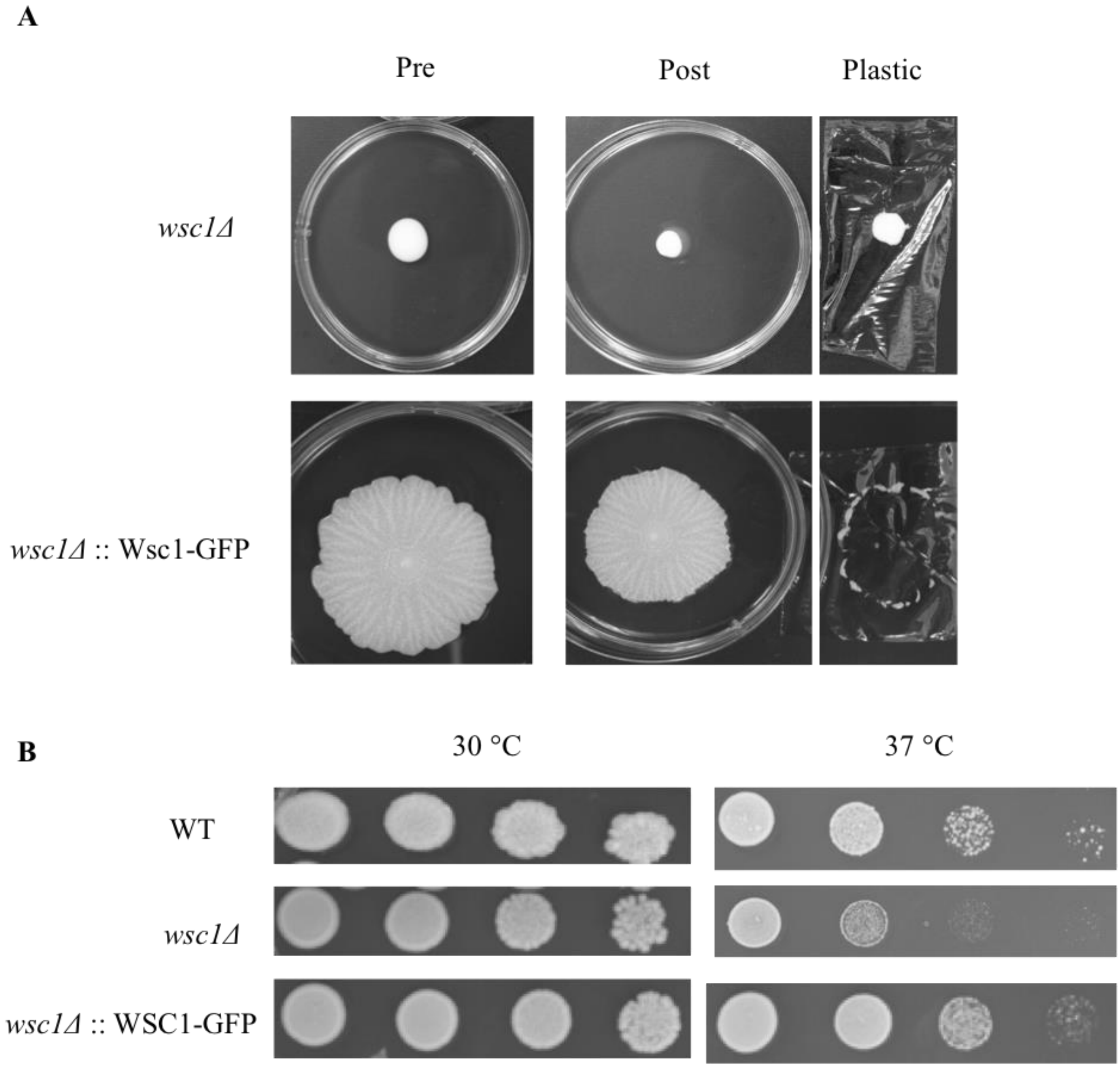

Figure S4 WSC1-GFP construct rescues mat formation defect and temperature sensitivity phenotypes of wsc1*Δ.*
(A) The mat formation phenotype and behavior in the overlay adhesion assay was assessed for the wild-type, *wsc1Δ,* and *wsc1Δ::WSC1-GFP* strains. (B) Growth of the strains was assessed after 48hrs at 37˚C.
